# Cell type-specific roles of PAR1 in Coxsackievirus B3 infection

**DOI:** 10.1038/s41598-021-93759-8

**Published:** 2021-07-12

**Authors:** Michael F. Bode, Clare M. Schmedes, Grant J. Egnatz, Vanthana Bharathi, Yohei M. Hisada, David Martinez, Tomohiro Kawano, Alice Weithauser, Leah Rosenfeldt, Ursula Rauch, Joseph S. Palumbo, Silvio Antoniak, Nigel Mackman

**Affiliations:** 1 Division of Cardiology, Department of Medicine, UNC McAllister Heart Institute, University of North Carolina at Chapel Hill, Chapel Hill, NC USA; 2 Division of Cardiology, Department of Medicine, Massachusetts General Hospital and Harvard Medical School, Boston, MA USA; 3 Division of Cardiology, Department of Medicine, Lahey Hospital & Medical Center, Burlington, MA USA; 4 Division of Hematology, Department of Medicine, UNC Blood Research Center, University of North Carolina at Chapel Hill, 116 Manning Drive CB 7035, 8004B Mary Ellen Jones Building, Chapel Hill, NC 27599 USA; 5 CharitéCentrum 11 Cardiovascular Diseases, Charité – Universitätsmedizin Berlin, Campus Benjamin Franklin, Berlin, Germany; 6 Cancer and Blood Disease Institute, Cincinnati Children’s Hospital Medical Center and the University of Cincinnati College of Medicine, Cincinnati, OH USA; 7 Department of Pathology and Laboratory Medicine, UNC Blood Research Center, UNC McAllister Heart Institute, University of North Carolina at Chapel Hill, Chapel Hill, NC USA

**Keywords:** Coagulation system, Toll-like receptors, Viral infection, Infection

## Abstract

Protease-activated receptor 1 (PAR1) is widely expressed in humans and mice, and is activated by a variety of proteases, including thrombin. Recently, we showed that PAR1 contributes to the innate immune response to viral infection. Mice with a global deficiency of PAR1 expressed lower levels of CXCL10 and had increased Coxsackievirus B3 (CVB3)-induced myocarditis compared with control mice. In this study, we determined the effect of cell type-specific deletion of PAR1 in cardiac myocytes (CMs) and cardiac fibroblasts (CFs) on CVB3-induced myocarditis. Mice lacking PAR1 in either CMs or CFs exhibited increased CVB3 genomes, inflammatory infiltrates, macrophages and inflammatory mediators in the heart and increased CVB3-induced myocarditis compared with wild-type controls. Interestingly, PAR1 enhanced poly I:C induction of CXCL10 in rat CFs but not in rat neonatal CMs. Importantly, activation of PAR1 reduced CVB3 replication in murine embryonic fibroblasts and murine embryonic cardiac myocytes. In addition, we showed that PAR1 reduced autophagy in murine embryonic fibroblasts and rat H9c2 cells, which may explain how PAR1 reduces CVB3 replication. These data suggest that PAR1 on CFs protects against CVB3-induced myocarditis by enhancing the anti-viral response whereas PAR1 on both CMs and fibroblasts inhibits viral replication.

## Introduction

Protease-activated receptor 1 (PAR1) is a member of a small family of seven membrane spanning G-protein coupled receptors (GPCRs) that are activated by proteolytic cleavage by a variety of proteases^1–5^. PARs allow cells to sense changes in the extracellular environment, such as activation of the coagulation cascade. Indeed, thrombin is a potent activator of PAR1^6^. PAR1 is widely expressed in humans and mice, and is the major thrombin receptor on human platelets^7^. Human platelets express PAR1 and PAR4 whereas mouse platelets PAR3 and PAR4^8^. In the heart, PAR1 is expressed by both cardiac myocytes (CMs) and cardiac fibroblasts (CFs)^9,10^. PAR1 has been shown to contribute to hypertrophy of CMs and proliferation of CFs^9–11^. Activation of PAR1 also increases cardiac injury and remodeling after ischemia–reperfusion injury^11,12^.

The innate immune system is rapidly activated in response to pathogens^13^. Infections are detected by a family of receptors that recognize pathogen-associated patterns^13^. For instance, toll-like receptor 3 (TLR3) recognizes double-stranded RNA that is generated during the replication of single-stranded RNA viruses^14,15^. Polyinosinic:polycytidylic acid (poly I:C) is a double-stranded RNA mimetic that is used to activate TLR3. Activation of TLR3 induces the expression of interferons (IFNs) and chemokines, such as CXCL10, that coordinate the early immune response to viral infection^5,14–16^. Cardiotropic viruses, such as the single-stranded RNA(+) virus Coxsackievirus B3 (CVB3), cause myocarditis^17–19^. Interestingly, TLR3 deficiency is associated with an increased viral load after infection with CVB3 or CVB4^20–24^. Similarly, IFN-β- and CXCL10-deficient mice are more susceptible to CVB3 infection than wild-type mice^25,26^. Furthermore, CVB3-induced myocarditis in mice and humans is reduced by IFN-α and IFN-β^24,27–29^.

Recently, we showed that PAR1 plays a role in CVB3 infection in mice^5,30^. We found that PAR1^-/-^ mice expressed lower levels of CXCL10 in the early phase of CVB3 infection that resulted in an increased viral load, inflammation and cardiac injury a later phase after infection^5^. In vitro studies with murine embryonic (me) CFs demonstrated that PAR1 activation enhanced poly I:C activation of the p38 MAPK pathway and induction of CXCL10 expression^5^. These data indicated that PAR1 on meCFs contributes to the TLR3-dependent innate response to CVB3 infection. CVB3 can infect and replicate in both CMs and CFs, although replication is higher in CF^31^. In addition, CFs infected with CVB3 express inflammatory mediators that aggravated myocarditis^31^. Importantly, CVB3 uses autophagy to increase its replication^32^.

In this study, we determined the effect of cell type-specific deletion of PAR1 in either CFs or CMs on CVB3-induced myocarditis. In addition, we analyzed the effect of PAR1 in CVB3 replication in murine embryonic fibroblasts (MEFs) and meCMs. Finally, we measured the effect of PAR1 activation on autophagy in MEFs and rat H9c2 cells.

## Material and methods

### Mice

To delete PAR1 in CMs (PAR1ΔCM), we used mice expressing the Cre recombinase under the control of the myosin light chain 2v (Mlc2v) promoter were purchased from Dr. Chien^33^. To delete PAR1 in CFs (PAR1ΔCF), we used mice expressing Cre recombinase under the transcription factor 21 (TCF21) obtained from Dr. Tallquist^34^. Female PAR1^fl/fl^ mice^35,36^ were crossed with male Cre-expressing mice to generate PAR1^fl/fl^;Mlc2v^Cre^ and PAR1^fl/fl^;TCF21^Cre-ERT2^. To activate TCF21^Cre-ERT2^ expression^34,37^ in PAR1^fl/fl^;TCF21^Cre-ERT2^ mice, 6 week old mice were gavaged with 2 mg of tamoxifen (Sigma-Aldrich, St. Louis, MO, USA) for 5 consecutive days and then mice used 5 days later. PAR1^fl/fl^ mice treated with tamoxifen were used as controls. In addition, we used wild-type (control) mice and mice with a global PAR1 deletion (ΔPAR1)^38^. All mice strains were on a C57Bl/6 J background. The studies were performed and approved in accordance with the guidelines of the Institutional Animal Care and Use Committee of the UNC-Chapel Hill and complied with National Institutes of Health and ARRIVE guidelines^39^.

### Virus infection and histology

Male mice were infected at 6–8 weeks of age intraperitoneal (i.p.) with 10^5^ plaque forming units (pfu) CVB3 (cardiotropic Nancy strain)^5,16,40^. Hearts were fixed in 10% formalin and embedded in paraffin (Sigma-Aldrich). Sections from paraffin-embedded sections were cut and stained with H&E. Area of cellular infiltration was calculated from whole cardiac tissue area on H&E stained tissue sections by 2 blinded investigators as described previously^5^. For macrophage staining, slides were blocked with 5% Non-fat skim milk (Applichem, Council Bluffs, IA, USA) in phosphate-buffered saline (Thermo Fisher Scientific, Waltham, MA, USA) for 1 h at room temperature. Endogenous avidin and biotin were blocked with avidin solution and biotin solution, respectively (Vector Laboratories, Burlingame, CA, USA). Sections were incubated overnight at 4 °C with 0.4 μg/mL rabbit anti-mouse Iba1 antibody (FUJIFILM Wako Chemicals, Osaka, Japan, CAT#019-19741). Sections were washed and incubated with biotinylated goat anti-rabbit antibody (Vector Laboratories, CAT# BA-1000) for 1 h at room temperature. Avidin-peroxidase complex solution (Vector Laboratories, CAT# PK-6100) was added and Iba-1 staining (brown) was detected using the DAB substrate (Agilent Technology, Santa Clara, Ca, USA, CAT#K3468). Sections were counterstaining with hematoxylin (Thermo Fisher Scientific) for 1 min. Sections were evaluated with a 1 to 5 scale by two blinded investigators.

### Measurement of CVB3 genomes and inflammatory mediators

Total RNA was prepared from heart and reverse transcribed into cDNA and levels of CVB3 genomes, PAR1, TNF-α and IL-6 mRNA measured by RT-PCR^5,30^. We used the following probe sets from Integrated DNA Technologies (Coralville, IA, USA): PAR1 forward, 5’-GGCGCTTGCTGATCGTC-3’; PAR1 reverse CGTAGCATCTGTCCTCTCTGA-3’; PAR1 probe, 5’-FAM-CGCGTCCCTATGAG-TAMRA-3’ and CVB3 forward, 5’-CCCTGAATGCGGCTAATCC-3’; CVB3 reverse, 5’-ATTGTCACCATAAGCAGCCA-3’; CVB3 probe, 5’-FAM-TGCAGCGGAACCG-TAMRA-3’. Variations in input RNA levels and reaction efficiency was corrected against levels of hypoxanthine guanine phosphoribosyltransferase (HPRT) from Integrated DNA Technologies (Mm.PT.39a.22214828). We used the following TaqMan probes sets from Applied Biosystems (Foster City, CA, USA): IL-6 (Mm99999064_m1) and TNF-α (Mm00443259_g1). Variations in input RNA levels and reaction efficiency was corrected against 18S rRNA (4310893E, Applied Biosystems). All RT-PCR reactions were performed with the SSoFast Advanced Universal Supermix in a Bio-Rad cycler (Bio-Rad Laboratories, Hercules, CA, USA) as described elsewhere^36,41^.

### Measurement of CVB3 titers

Hearts were homogenized in minimum essential media, frozen and thawed 5 times and centrifuged to pellet cellular debris. The supernatant was serial diluted and added to plates of HeLa cells in replicates of 6. Virus titers were determined after 2–3 days of incubation^5,16^.

### Measurement of CXCL10

Levels of CXCL10 protein in the culture supernatant of cardiac cells were measured by ELISA (R&D Systems, Minneapolis, MN, USA)^5,30^.

### Isolation of embryonic and neonatal cardiac myocytes, cardiac fibroblasts and murine embryonic fibroblasts

Hearts of wild-type and ΔPAR1 embryos (embryonic day [E]14.5) were isolated and incubated overnight in 0.25% trypsin/EDTA (Sigma-Aldrich). Cell suspensions were plated for 90 min to allow adherence of the CFs^5,16,41^. Non-adherent meCMs were isolated by adherence overnight. MEFs were derived from wild-type embryos (E14.5) as described^41^. In addition, rat neonatal (rn) CFs and CMs were isolated from 1 day old rat pups as described^2^. The addition of 0.1 mM BrdU to the CM culture maintenance medium prevented the proliferation of any contaminating CFs. Equal distribution of both sexes within each litter was assumed. Cells were stimulated with Poly I:C (25 µg/mL, γ-radiated; Sigma-Aldrich), thrombin (IIa, 5–10 nM, human α-thrombin, Enzyme Research Laboratories, South Bend, IN, USA) or PAR1 agonist peptide (PAR1 AP) (TFLLR, 200 µM, Abgent, San Diego, CA, USA) in serum-free media (SFM)^5,30,41^.

### Measurement of autophagy

To analyse autophagic flux, wild-type (control) or ΔPAR1 MEFs were seeded the day before in growth media (GM). The next day, GM was replaced with either serum-free media (SFM) alone or SFM supplemented with chloroquine (CQ, 10 µM).

Rat H9c2 CMs (ATCC, Manassas, VA, USA)^42^ were infected with CVB3 (MOI 5) for 30 min in SFM. After a 30 min incubation period, media was replaced with SFM with or without thrombin (10 nM) for 24 h. After 6 h or 24 h, cells were lysed in RIPA buffer and subjected to Western blot analyses as described^5,41^. Autophagic flux was determined using an anti-light chain 3A (LC3A) antibody (1:1000, #PM036, MBL International Corporation, Woburn, MA, USA), which detects LC3-I and LC3-II.

To analyse the effect of thrombin stimulation on autophagy, wild-type MEFs or H9c2 CMs were seeded in GM. The next day, cells were either further cultured in GM or in SFM with or without 10 nM thrombin for 24 h. Total cell lysates were subjected to Western blot analyses for LC3A, p62 (1:1000, #PM045, MBL International Corporation) and ZBTB16 (1:1000, ab39354, Abcam, Cambridge, MA, USA)^43^. Loading differences were normalized to β-actin protein levels (Abgent). Images of the Western blots were obtained with an Odyssey LI-COR imager (LI-COR Bioscience, Lincoln, NE, USA) with a 700 and 800 nm filter^5^. Specific band intensities were determined with ImageJ (National Institutes of Health, Bethesda, MA, USA)^5^.

### Statistics

All statistical analyses were performed in GraphPad Prism 9.1 (GraphPad Software Inc., La Jolla, CA, USA). Data are represented as mean ± SEM. For 2-group comparison of normally distributed data, the 2-tailed Student’s* t* test was used. For multiple-group comparison, normally distributed data were analyzed by 1- or 2-way ANOVA tests and were Bonferroni-corrected for repeated measures over time. *P*-value ≤ 0.05 was regarded as significant.

## Results

### Cell-specific deletion of PAR1 in the heart using cardiac fibroblast and cardiac myocyte specific Cre expression

**Figure 1:**
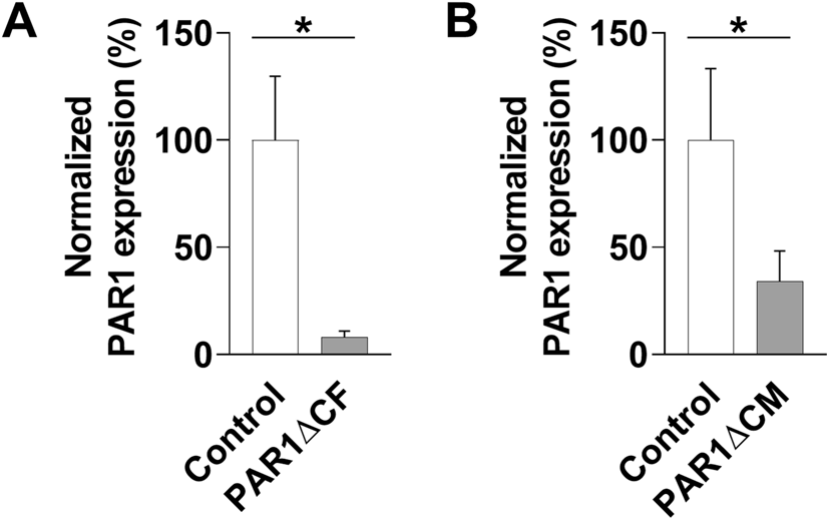
PAR1 expression in hearts of PAR1ΔCF and PAR1ΔCM mice. PAR1 mRNA expression in (**A**) uninfected hearts of PAR1ΔCF (PAR1^fl/fl^;TCF21^ETR2Cre^) mice and (**B**) PAR1ΔCM (PAR1^fl/fl^;Mlc2v^Cre^) mice and their respective control (PAR1^fl/fl^) littermates was analyzed by RT-PCR. PAR1 mRNA expression was normalized to HPRT mRNA expression. Data (mean ± SEM) of control littermates were set to 100% and analyzed by Student’s t test. *P < 0.05. (**A**: N = 5–6, **B**: N = 9–10).

To analyze the deletion rate of PAR1 in hearts from the two transgenic lines, we quantified levels of PAR1 mRNA expression in hearts of uninfected PAR1ΔCF, PAR1ΔCM mice and their respective control littermates by RT-PCR. Expression of TCF21-promotor controlled Cre recombinase after tamoxifen-treatment for 5 days resulted in significantly reduced expression of PAR1 in hearts of PAR1ΔCF mice compared to the tamoxifen-treated control mice (Fig. 1A). In addition, cardiomyocyte-specific Cre recombinase expression significantly reduced PAR1 mRNA expression in hearts of PAR1ΔCM mice compared to control littermates (Fig. 1B).

### Mice with PAR1 deleted in either cardiac fibroblasts or cardiac myocytes have increased levels of CVB3 genomes, inflammatory mediators and inflammatory cells in their hearts after CVB3 infection

**Figure 2:**
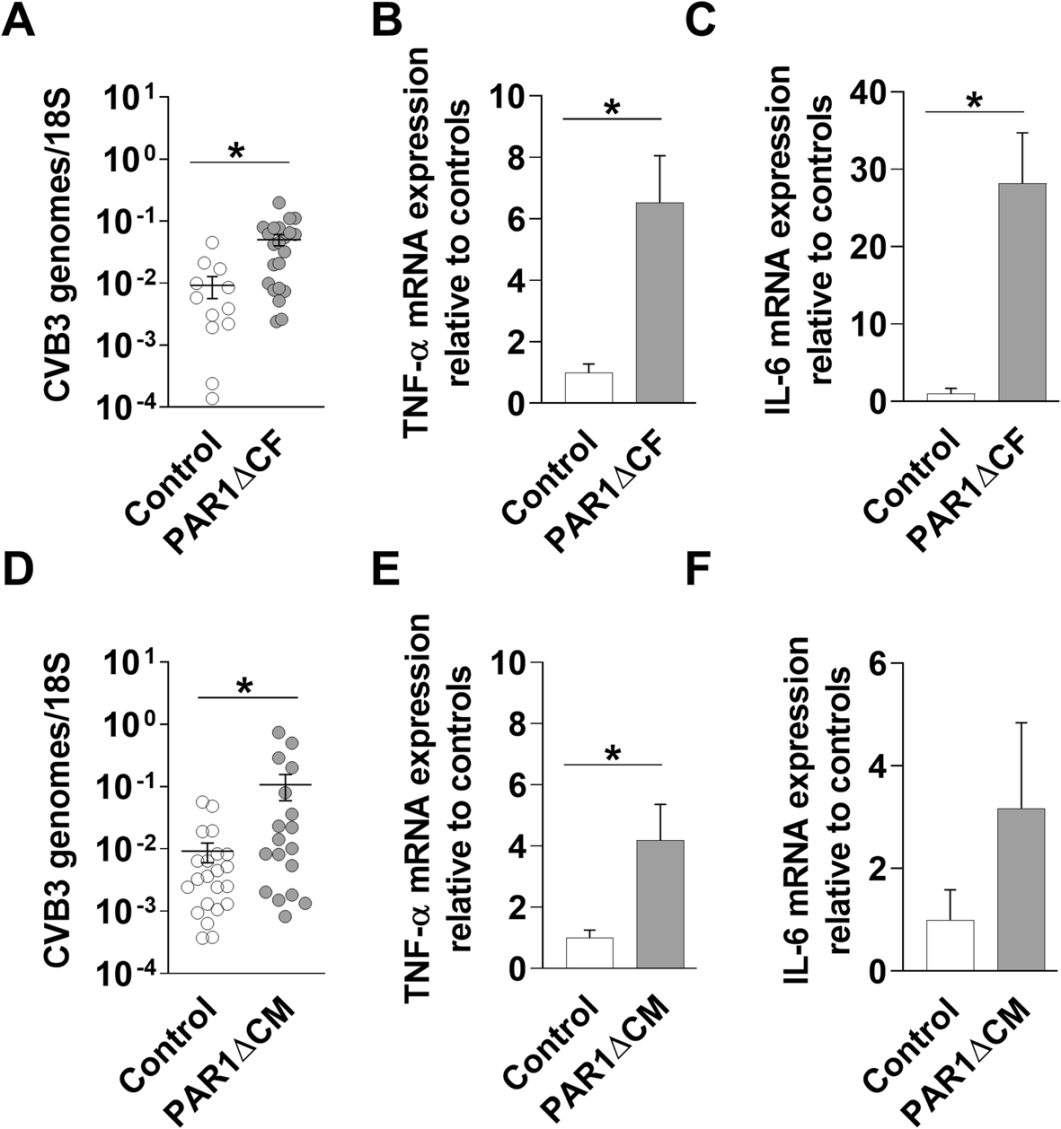
Mice with PAR1 deficiency in cardiac fibroblasts or cardiac myocytes have increased levels of CVB3 genomes and inflammatory mediators. Levels of CVB3 genomes (**A**,**D**), TNF-α mRNA (**B**,**E**) and IL-6 mRNA (**C**,**F**) were increased in the hearts of PAR1ΔCF (PAR1^fl/fl^;TCF21^ETR2Cre^) mice and PAR1ΔCM (PAR1^fl/fl^;Mlc2v^Cre^) mice compared with infected control (PAR1^fl/fl^) littermates 8 days after CVB3 infection. Data are shown as mean ± SEM and analyzed by Student’s *t* test. *P < 0.05. (N = 7–22).

**Figure 3:**
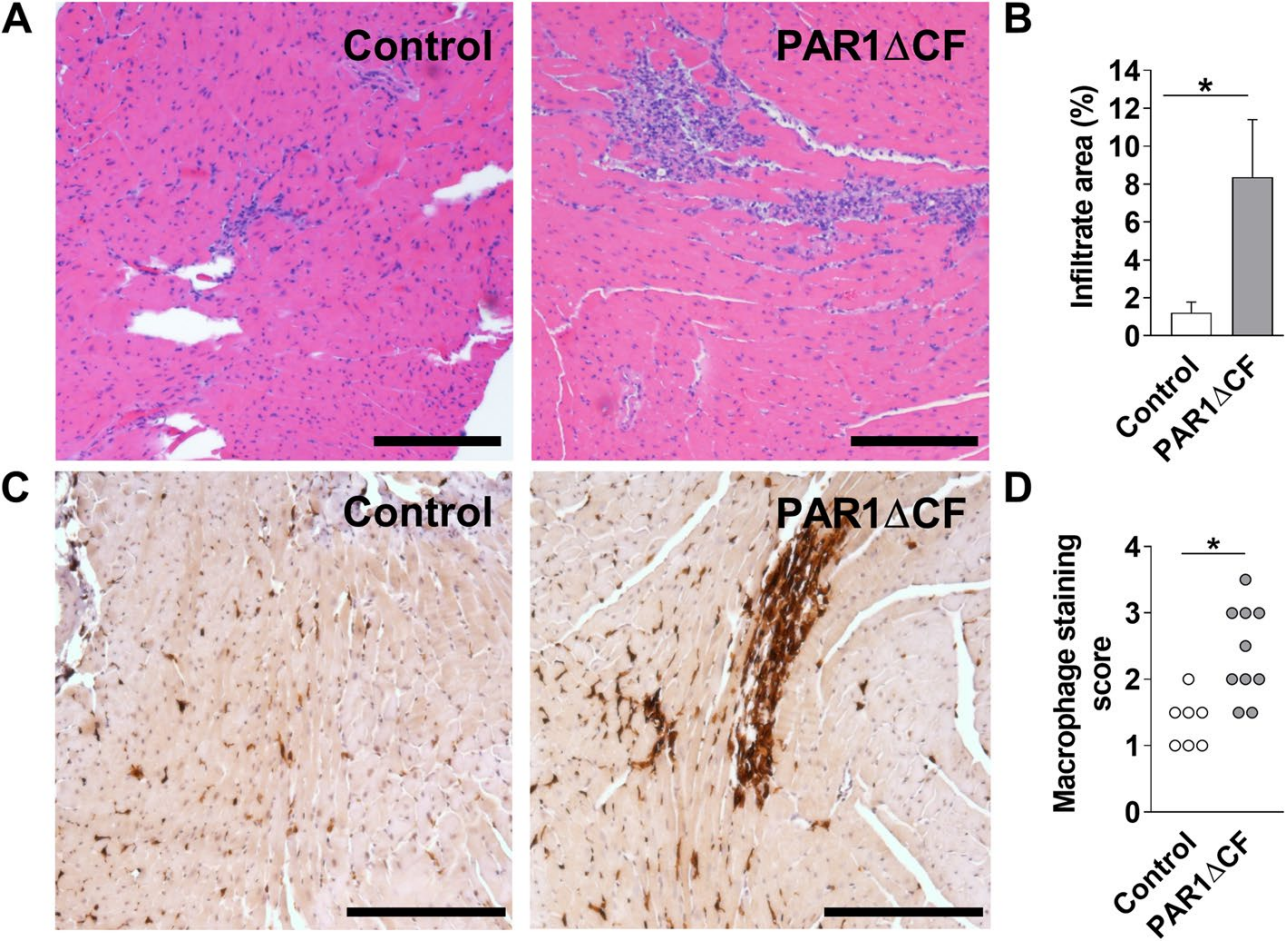
Mice with PAR1 deficiency in cardiac fibroblasts have increased CVB3-induced myocarditis. Representative H&E staining (**A**) and quantified immune cell infiltration area (**B**), representative macrophage staining (**C**) and macrophage staining score of heart sections form PAR1ΔCF (PAR1^fl/fl^;TCF21^ETR2Cre^) mice and control (PAR1^fl/fl^) littermates 8 days after CVB3 infection. Bar = 200 µm. Data are mean ± SEM and analyzed by Student’s *t* test. *P < 0.05. (**B**: N = 7–10; **D**: N = 8–10).

**Figure 4:**
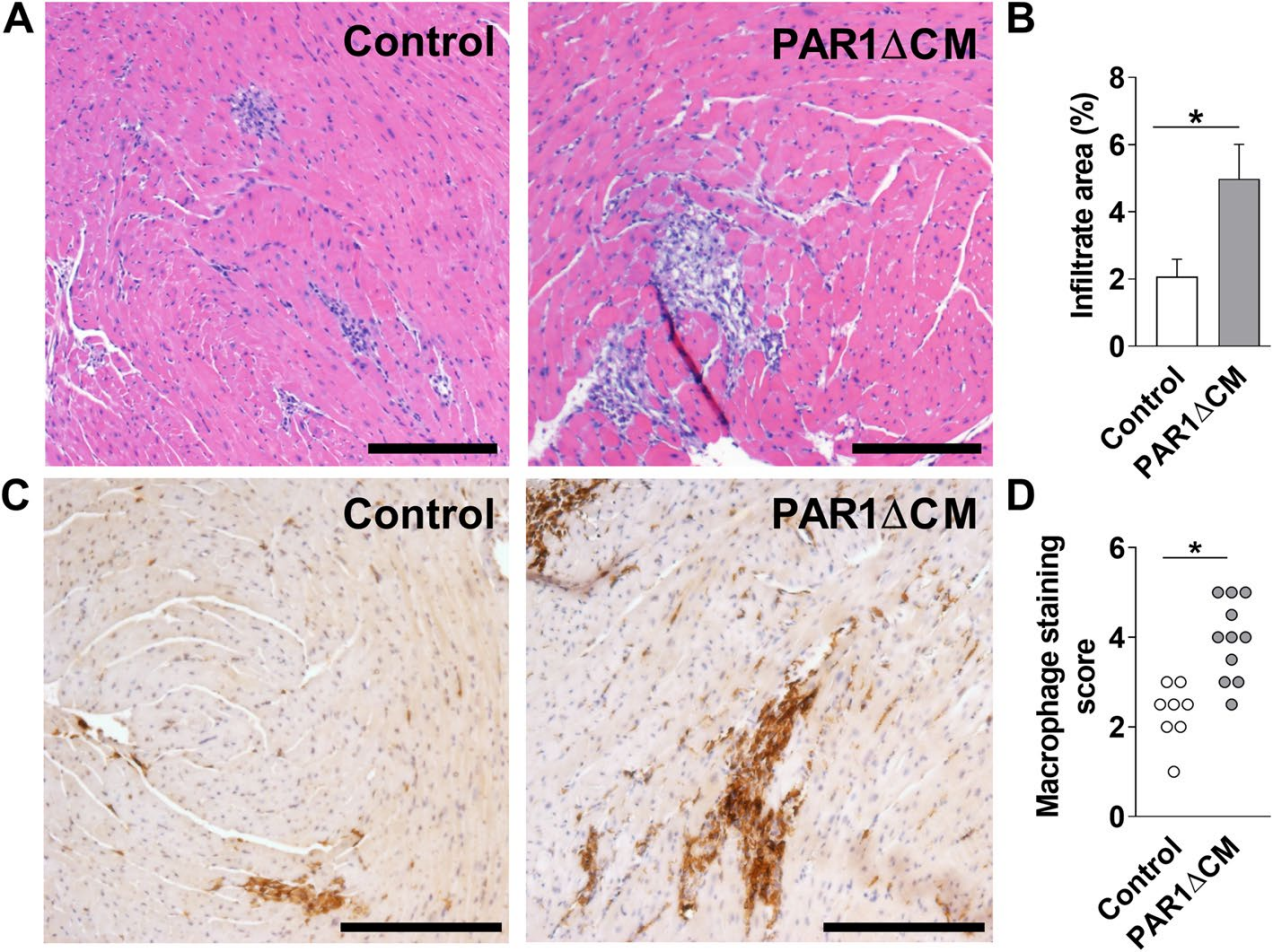
Mice with PAR1 deficiency in cardiac myocytes have increased CVB3-induced myocarditis. Representative H&E staining (**A**) and quantified immune cell infiltration area (**B**), representative macrophage staining (**C**) and macrophage staining score of heart sections form from PAR1ΔCM (PAR1^fl/fl^;Mlc2v^Cre^) mice and control (PAR1^fl/fl^) littermates 8 days after CVB3 infection. Bar = 200 µm. Data are mean ± SEM and analyzed by Student’s *t* test. *P < 0.05. (**B**: N = 18–22; **D**: N = 8–11).

We determined the effect of deleting PAR1 in either CFs or CMs on CVB3-induced myocarditis. PAR1ΔCF mice exhibited significantly increased CVB3 genome levels, TNF-α mRNA and IL-6 mRNA expression in their hearts 8 days after infection compared with controls (Fig. 2A–C). Similarly, PAR1ΔCM mice had a significant increase in CVB3 genomes and TNF-α mRNA expression in their hearts compared with control mice 8 days after infection (Fig. 2D–F). PAR1ΔCF and PAR1ΔCM mice also had significantly higher levels of inflammatory infiltrates and macrophages in their hearts compared with control mice 8 days after CVB3 infection (Figs. 3 and 4).

### Effect of PAR1 activation on poly I:C induction of CXCL10 in cardiac fibroblasts and cardiac myocytes

**Figure 5:**
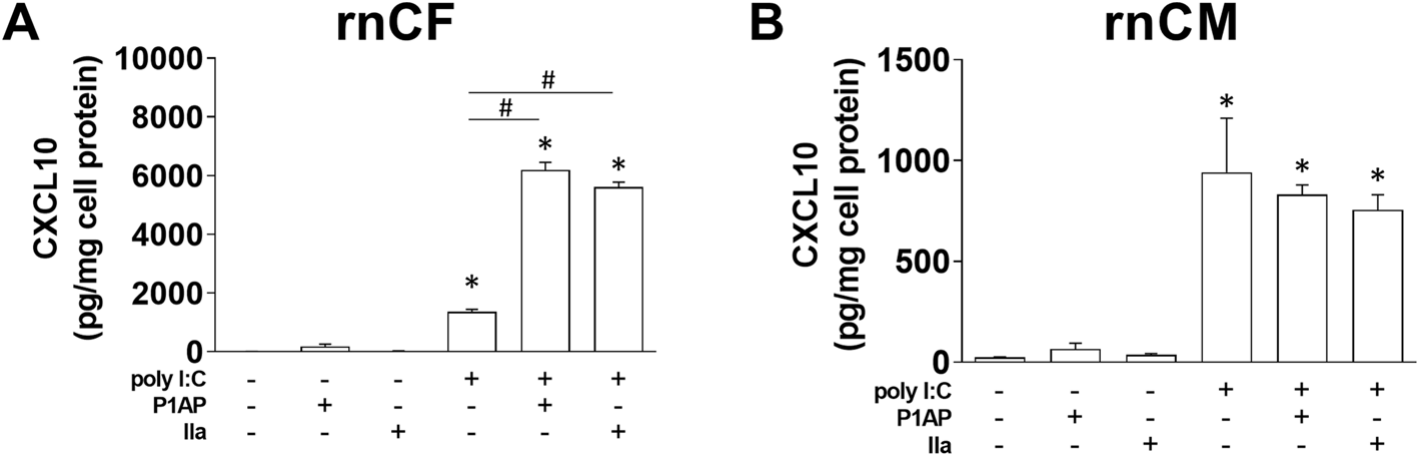
Effect of PAR1 activation on poly I:C induction of CXCL10 expression in rat neonatal cardiac fibroblasts and cardiac myocytes. Rat neonatal cardiac fibroblasts (**A**) and cardiac myocytes (**B**) were stimulated with either poly I:C (25 µg/mL), PAR1 agonist peptide (TFLLR, 200 µM), thrombin (IIa, 5 nM) or combinations of agonists for 8 h. Levels of CXCL10 in the supernatant were measure by ELISA in triplicate. Data are mean ± SEM and analyzed by 1-way ANOVA with Bonferroni post-test. *P < 0.05 vs unstimulated control, ^#^P < 0.01 vs poly I:C treated sample. (N = 3).

We recently showed that PAR1 activation enhanced poly I:C induction of CXCL10 expression in meCFs^5^. In this study, we used rnCFs and rnCMs because larger quantities of cells can be isolated. We confirmed that either thrombin or a PAR1 AP enhanced poly I:C induction of CXC10 expression in rnCFs (Fig. 5A). In contrast, treatment of rnCMs with either thrombin or PAR1 agonist peptide did not enhance poly I:C induction of CXC10 expression (Fig. 5B).

### Effect of PAR1 on adhesion and uptake of CVB3 in cardiac myocytes

For CVB3 adhesion experiments, serum-containing GM was removed and the rnCMs were infected with CVB3 multiplicity of infection (MOI) of 1 under serum-free conditions and incubated for 30 min on ice. Next, rnCMs were washed twice with ice-cold PBS and total RNA was isolated. Virus genomes were measured by RT-PCR^44^. We found that activation of PAR1 with an agonist peptide did not affect adhesion of CVB3 (data not shown).

To analyse CVB3 uptake, rnCMs cells were incubated with CVB3 (MOI 1) under serum-free conditions for 30 min on ice. Next, rnCMs were washed and incubated in SFM for 1 h at 37 °C. Trypsin was added to the cells for 1.5 min to remove the remaining virus from the cell surface. CMs were washed twice with PBS, total RNA was isolated and virus genomes measured by RT-PCR^44^. Activation of PAR1 with an agonist peptide did not affect uptake of CVB3 (data not shown).

### PAR1 reduces CVB3 replication in murine embryonic fibroblasts and murine embryonic cardiac myocytes

**Figure 6:**
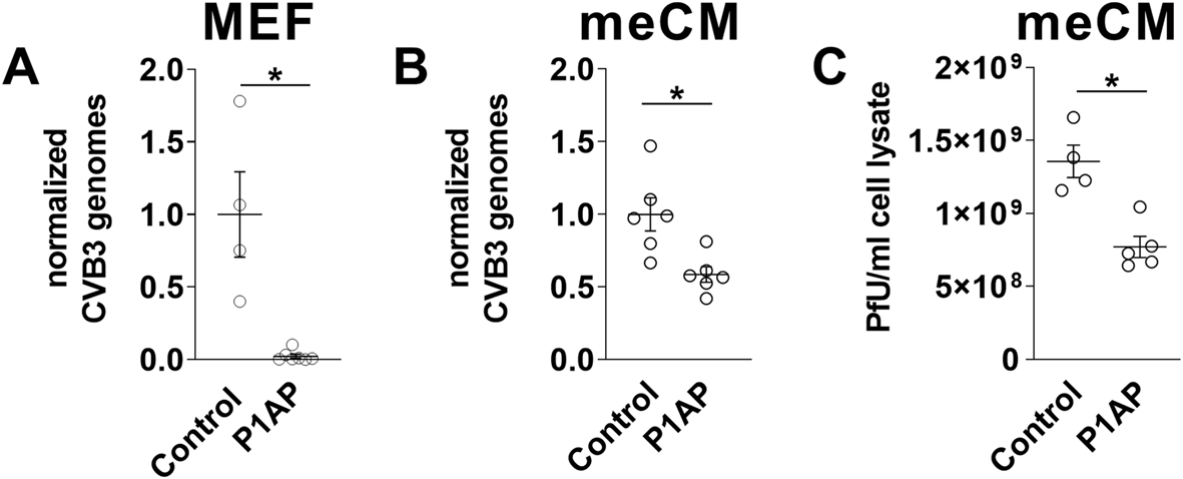
Effect of PAR1 activation on CVB3 replication in mouse embryonic fibroblasts and myocytes. (**A**) Wild-type (WT) mouse embryonic fibroblasts were infected with CVB3 (multiplicity of infection [MOI] 1) and stimulated with PAR1 agonist peptide (P1AP) (200 µM) to analyze CVB3 replication 6 h after infection. (**B**) Murine embryonic cardiac myocytes from WT mice were infected with CVB3 (MOI 1) with or without PAR1AP (200 µM) stimulation to analyze the effect of PAR1 activation on virus genome levels 6 h after infection. (**C**) Levels of active CVB3 virus was analyzed by plaque assay in WT murine embryonic cardiac myocytes infected with CVB3 (MOI 1) and stimulated with/ without P1AP (200 µM) for 24 h. Virus genome levels (**A**–**C**) were analyzed by real-time PCR. Virus genomes were normalized to expression of hypoxanthine guanine phosphoribosyltransferase mRNA and set was set to 1 for CVB3 infected control cells of each genotype. Data are mean ± SEM and analyzed by Student’s *t* test. *P < 0.05.

Next, we determined if PAR1 stimulation affects CVB3 replication. GM was aspirated from the cells and CVB3 solution (MOI 1) was added to MEFs or meCMs. After 30 min at 37 °C the medium was aspirated, and fresh GM was added to the cells. Levels of CVB3 genomes were analysed 6 h after infection by RT-PCR and normalized using HPRT mRNA^5,30^. Active CVB3 virus was analysed after 24 h^5,16^. We found that PAR-1 AP stimulation reduced CVB3 genome levels in MEFs (Fig. 6A). In addition, we also observed significantly reduced levels of CVB3 genomes in meCMs treated with a PAR1 agonist peptide (Fig. 6B) indicating that PAR1 in CMs limits CVB3 replication. We did not observe a decrease in CVB3 infection of meCMs lacking PAR1 compared to wild-type cells (data not shown). Treatment of meCMs with a PAR1 AP also significantly reduced the number of CVB3 pfu (Fig. 6C).

### PAR1 activation reduces autophagy

**Figure 7:**
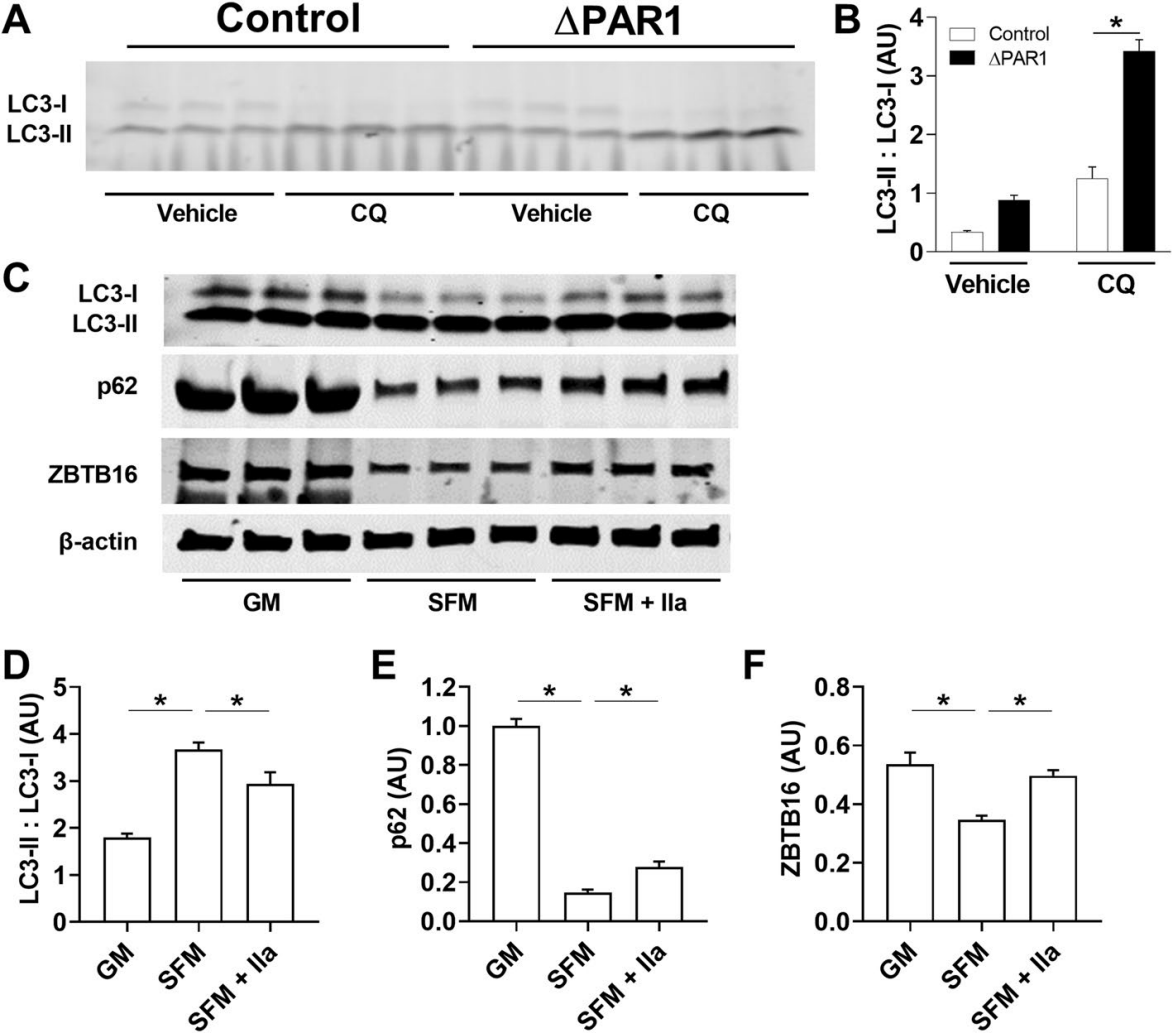
Effect of thrombin stimulation and PAR1 deficiency on autophagy. (**A**,**B**) Autophagy flux was determined by measuring levels of LC3-I and LC3-II levels by Western blotting in murine embryonic fibroblasts (MEFs) isolated from wild-type (white bars) and ΔPAR1 (black bars) embryos with or without 10 µM chloroquine (CQ) for 6 h under serum-free conditions. (**C**) Wild-type MEFs were cultured in serum containing growth media (GM), serum-free media (SFM) with or without thrombin (IIa, 10 nM) for 24 h. Levels of LC3-I/II, p62, ZBTB16 and actin were determined by Western blotting. Densitometry was performed to calculate LC3-II to LC3-I ratios and p62 and ZBTB16 protein expression. Levels of p62 and ZBTB16 were normalized to total β-actin protein levels (**D**–**F**). Data are mean ± SEM and analyzed by 1-way and 2-way ANOVA with Bonferroni post-test. *P < 0.05. (**B**: N = 3; **C**–**E**: N = 6).

CVB3 uses autophagy to increase its replication^32^. In addition, a recent publication showed that GPCR activation reduces autophagy^45^. We compared autophagic flux in wild-type and ΔPAR1 MEFs by analyzing the conversion of the cytosolic precursor form of microtubule-associated protein LC3-I to the autophagosome-localized, cleaved, and lipidated form LC3-II^43^. We found that MEFs isolated from ΔPAR1 exhibited an increased LC3-II/LC3-I ratio, which is an indicator for increased autophagic flux (Fig. 7A,B). We used the autophagy inhibitor CQ, which inhibits the last stage of autophagy by preventing protein degradation that leads to an accumulation of LC3-II. Again, ΔPAR1 cells exhibited an increase in autophagy flux in the presence of CQ compared with wild-type cells (Fig. 7A,B).

Next, we analyzed if thrombin stimulation of wild-type MEFs can reverse the autophagy induction during serum-starvation. First, we analyzed the changes in LC3-II/LC3-I ratios in MEFs cultured under serum and serum-free condition with or without thrombin stimulation. As expected, serum deprivation leads to autophagy induction seen as an increase in the LC3-II:LC3-I ratio (Fig. 7C,D). Thrombin stimulation significantly reduced the LC3-II/LC3-I ratio induced by serum starvation (Fig. 7C,D). Next, we investigated the changes in p62 protein levels (also known as Sequestosome 1, or SQSTM1, a substrate degraded by autophagy). Again, serum deprivation drastically reduced p62 levels in MEFs and thrombin stimulation resulted in significantly higher p62 levels compared to serum deprivation alone (Fig. 7C,E). Lastly, we analyzed the changes in ZBTB16 protein levels, also known as promyelocytic leukemia zinc finger (PLZF) or Zfp145, which was shown to be important for GPCR-mediated autophagy inhibition^45^. Serum deprivation caused a reduction in ZBTB16 in MEFs that was significantly reduced by thrombin (Fig. 7C,F).

**Figure 8:**
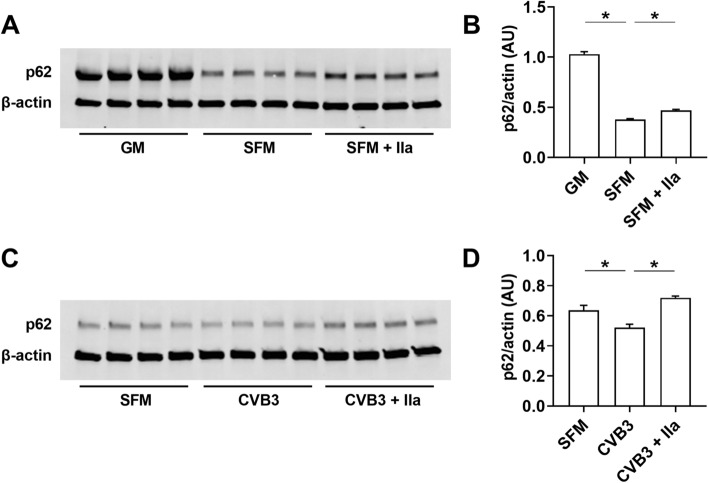
Thrombin reduces autophagy in serum-starved or CVB3 infected H9c2 cells. (**A**,**B**) Levels of p62 in H9c2 cells with or without serum deprivation in the presence or absence of thrombin (10 nM) for 24 h. Levels of p62 were normalized to β-actin protein levels. (**C**,**D**) Levels of p62 in H9c2 cells infected with CVB3 in the absence and presence of thrombin (10 nM) for 24 h. Levels of p62 were normalized to β-actin protein levels. Data are mean ± SEM and analyzed by 1-way ANOVA. *P < 0.05. (N = 4).

We found that thrombin significantly inhibited the loss of p62 expression induced by serum deprivation in the rat H9c2 cells (Fig. 8A,B). In addition, thrombin abolished the reduction on p62 in rat H9c2 cells infected with CVB3 (Fig. 8C,D).

## Discussion

In this study, we found that mice lacking PAR1 in either CFs or CMs had increased CVB3-induced myocarditis compared with controls, which indicated that PAR1 on these different cell types regulates viral infection. Previously, we have shown that PAR1 activation in CFs enhances poly I:C induction of IFN-β and CXCL10^5^. Here, we found that PAR1 activation did not enhance poly I:C induction of CXCL10 in CMs. However, we found that PAR1 activation on CMs reduced CVB3 replication in MEF and CMs. Furthermore, PAR1 expression and activation reduced serum deprivation and CVB3 infection-induced autophagy in MEFs and rat H9c2 CMs. This suggests that PAR1 enhances the anti-viral response to CVB3 infection and inhibits its replication by reducing autophagy.

We found higher levels of PAR1 mRNA in CFs compared to CMs (data not shown). The fact that PAR1 enhances CXCL10 expression in CFs but not CMs is not surprising. Although CFs express lower basal levels of IFN-β compared to CMs, CFs have a larger induction of IFN-β after virus infection compared to CMs^46,47^. In addition, activation of the IFN-β-dependent signaling pathway in CFs was found to be more important to restrict virus replication compared to CMs^46^. Together, this suggests that CFs are more reliant on the induction of this antiviral pathway than CMs after viral infection. Therefore, PAR1 enhancement of the IFN-β-dependent signaling pathway in CFs will have a greater impact on CVB3-induced myocarditis than its expression in CMs^46^.

In line with the notion of a cell-specific response to PAR1 activation, Sabri et al.^10^ showed that CFs exhibited a different intracellular signaling pattern compared to CMs after PAR1 stimulation. These differences were attributed to specific PAR1-dependent transactivation of the epidermal growth factor receptor (EGFR) via Src leading to enhanced p38 signaling in CFs^2,10^. PAR1 transactivation of EGFR is not observed in CMs^10^. Importantly, EGFR-dependent Src activity was required for effective TLR3-dependent immune responses^48^. Activated Src led to TLR3 phosphorylation leading to increased TRIF binding and subsequently enhanced TLR3:TRIF signaling^48^.

We found that PAR1 activation has no influence on CVB3 infectivity with regards to virus adhesion and uptake into CMs. CVB3 mainly uses the coxsackievirus adenovirus receptor (CAR) and its co-receptor decay-accelerating factor (DAF) to infect cells^19^. Moreover, CMs are the primary site of CVB3 replication in the heart^49^. Interestingly, it was shown that uptake of the enveloped DNA virus, herpes simplex virus 1, into cells is increased by PAR1-dependent signaling^50^. However, there was no PAR1-dependency in the infection of cells with another enveloped virus, influenza virus A^51^. The lack of a PAR1 effect on CVB3 infection of CMs suggests that PAR1 does not affect CVB3 interaction with CAR and/or DAF. The differences in PAR1-dependency on uptake of these three viruses is surprising since they all use endocytosis for cell entry.

Importantly, we found that PAR1 stimulation reduced CVB3 replication in MEFs and CMs. A hallmark of RNA( +) viruses, such as CVB3, is that they use host membrane structures for their replication^19,32^. After CVB3 infects cells it associates with intracellular membrane structures (multivesicular bodies, [MVB]) via endosomes, which supports virus replication^19^. While CVB3 initiates early endosomal/autophagic processes it is able to block the maturation and fusion with lysosomes inhibiting its own degradation^19^. Here, we showed that PAR1 deficiency was associated with reduced autophagic flux in MEFs as measured by an increase in the LC3-II:LC3-I ratio. We showed that MEFs without stimulation release PAR1 activating proteases, such as matrix metalloproteinases 13^2,5^, causing paracrine and/or autocrine PAR1-dependent autophagy inhibition in wild-type but not PAR1 deficient cells. Interestingly, an increase in the conversion of LC3-I to LC3-II and autophagic flux was shown to enhance CVB3 replication^52,53^. A recent study showed that GPCRs activation reduces autophagy by stabilizing ZBTB16 protein levels^45^. Serum starvation was shown to cause ZBTB16 proteasomal degradation due to increased glycogen synthase kinase-3 (GSK3) activity, which subsequently leads to autophagy induction^45^. We showed that serum starvation increases ZBTB16 degradation and autophagy induction in MEFs. Interestingly, PAR1 activation with thrombin was able to reduce autophagy induction as determined by a lower LC3-II:LC3-I ratio and higher p62 and ZBTB16 protein levels. In addition, autophagy induced by either serum deprivation or CVB3 infection of H9c2 cells was significantly reduced by thrombin. This might be due to the known effect of PAR1 activation on GSK3 inhibition^54^, which could lead to increased inhibition of autophagy^45^. A recent study showed that CVB3 protease 2A can reduce p62 levels by direct cleavage causing increased CVB3 replication^55^. Furthermore, overexpression of p62 restricted whereas p62 *knock-down* supported CVB3 replication in vitro^55^. Thrombin can also activate PAR4. However, we found that activation of PAR4 does not affect CVB3 replication in CMs^41^, which suggested that PAR1 but not PAR4 influences CVB3 life cycle in CMs possibly by maintaining p62 levels. Together, the negative effects of PAR1 on autophagy might be the reason we see reduced virus replication in wild-type cells stimulated with thrombin. Our observation of PAR1-dependent autophagy inhibition might explain certain protective phenotypes in ΔPAR1 mice, such as in cardiac ischemia–reperfusion injury^11^, chronic angiotensin-II infusion^56^ or doxorubicin induced heart failure^57^ where increased autophagy was shown to be protective^58–60^.

## Conclusion

In this study, we showed that deletion of PAR1 in either CFs or CMs was associated with increased CVB3-induced myocarditis. PAR1 activation enhanced poly I:C induction of in CFs but not CMs. PAR1 activation reduced CVB3 replication in MEFs and CMs. Finally, thrombin activation of PAR1 inhibited autophagy, which may explain its inhibitory effect on CVB3 replication.

## Supplementary Information

Supplementary Information.

## Abbreviations

CVB3: Coxsackievirus B3
IFN: Interferon
Mlc2v: Myosin light chain 2v
PCR: Polymerase chain reaction
PAR1: Protease-activated receptor 1
TCF21: Transcription factor 21
TLR3: Toll-like receptor 3
