# Supplementary material for: Cell type-specific roles of PAR1 in Coxsackievirus B3 infection
Source: Sci Rep. 2021 Jul 12;11:14264. doi: 10.1038/s41598-021-93759-8 (PMC8275627; doi:10.1038/s41598-021-93759-8)
Supplement: Supplementary file 1 — Supplementary Information. [file 41598_2021_93759_MOESM1_ESM.pdf]

Cell type-specific roles of PAR1 in Coxsackievirus B3 infection

Michael F Bode[1,2,3], Clare M Schmedes[4], Grant J Egnatz[4], Vanthana Bharathi[4], Yohei M Hisada[4], David Martinez[4], Tomohiro Kawano[4], Alice Weithauser[5], Leah Rosenfeldt[6], Ursula Rauch[5], Joseph S Palumbo[6], Silvio Antoniak[7], Nigel Mackman[4]

[1]UNC McAllister Heart Institute, Division of Cardiology, Department of Medicine, University of North Carolina at Chapel Hill, Chapel Hill, North Carolina, USA

[2]Division of Cardiology, Department of Medicine, Massachusetts General Hospital and Harvard Medical School, Boston, Massachusetts, USA

[3]Lahey Hospital & Medical Center, Division of Cardiology, Department of Medicine, Burlington, Massachusetts, USA

[4]UNC Blood Research Center, Division of Hematology, Department of Medicine, University of North Carolina at Chapel Hill, Chapel Hill, North Carolina, USA

[5]CharitéCentrum 11 Cardiovascular Diseases, Charité – Universitätsmedizin Berlin, Campus Benjamin Franklin, Berlin, Germany

[6]Cancer and Blood Disease Institute, Cincinnati Children's Hospital Medical Center and the University of Cincinnati College of Medicine, Cincinnati, Ohio, USA

[7]UNC Blood Research Center, UNC McAllister Heart Institute, Department of Pathology and Laboratory Medicine, University of North Carolina at Chapel Hill, Chapel Hill, North Carolina, USA

Corresponding Author:
Nigel Mackman, Ph.D.
Department of Medicine
116 Manning Drive CB 7035
8004B Mary Ellen Jones Building
University of North Carolina at Chapel Hill Chapel Hill, NC 27599, USA
Email: nmackman@med.unc.edu
Tel: (919) 843-3961
Fax: (919) 966-6012

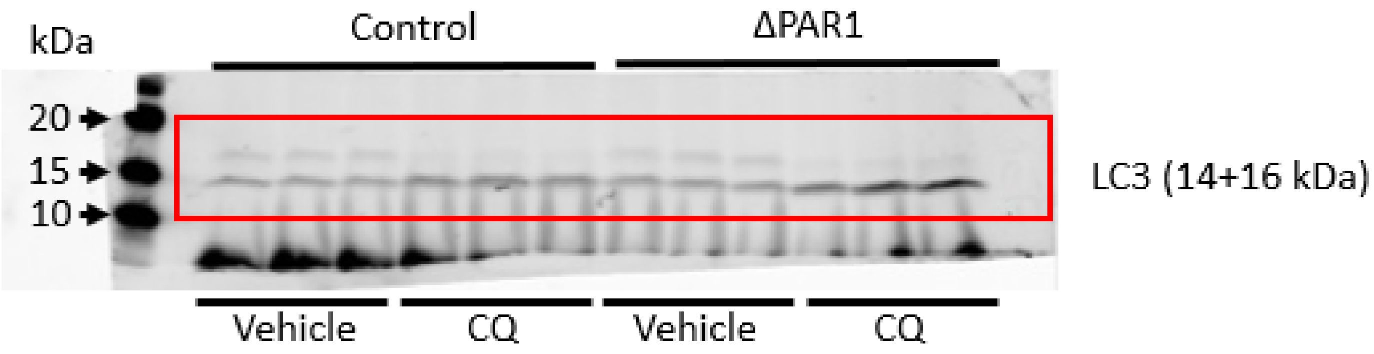

Supplemental Figure 1: Uncut membrane for figure 7A - Effect of thrombin stimulation and PAR1 deficiency on autophagy.

Autophagy flux was determined by measuring levels of LC3-I and LC3-II levels by Western blotting in wild-type and ΔPAR1 murine embryonic fibroblasts with or without 10 μM chloroquine (CQ) for 6 hours under serum-free conditions.

Box indicates cropped membrane as shown in figure 7A of the main text.

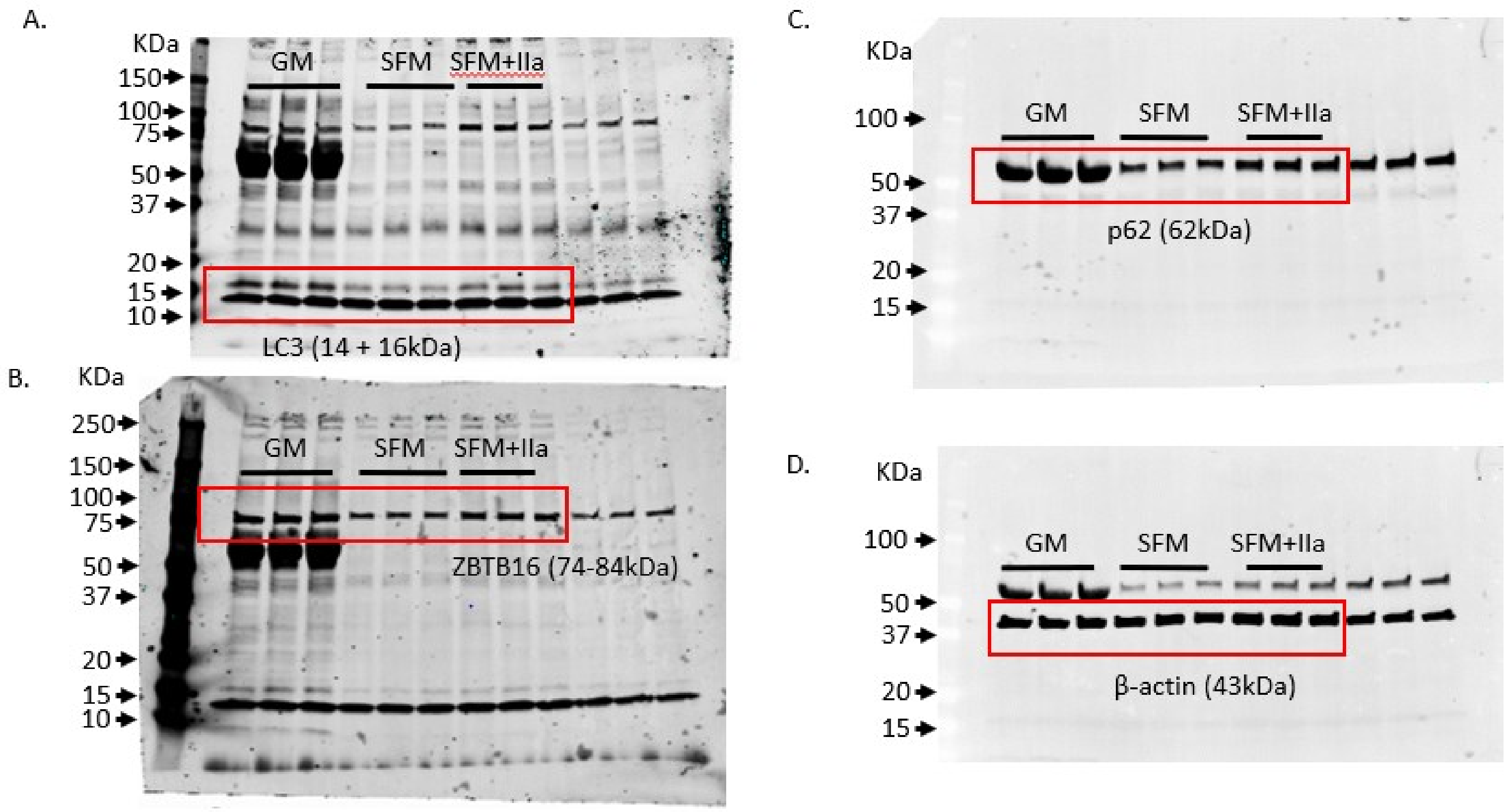

Supplemental Figure 2: Uncut membrane for figure 7C - Effect of thrombin stimulation and PAR1 deficiency on autophagy.

Wild-type murine embryonic fibroblasts were cultured in serum containing growth media (GM), serum-free media (SFM) with or without thrombin (IIa, 10 nM) for 24 hours. Specific Western blot bands for LC3-I/II **(A)**, ZBTB16 **(B)**, p62 **(C)** and β-actin **(D)**. Box indicates cropped membrane as shown in figure 7C of the main text.

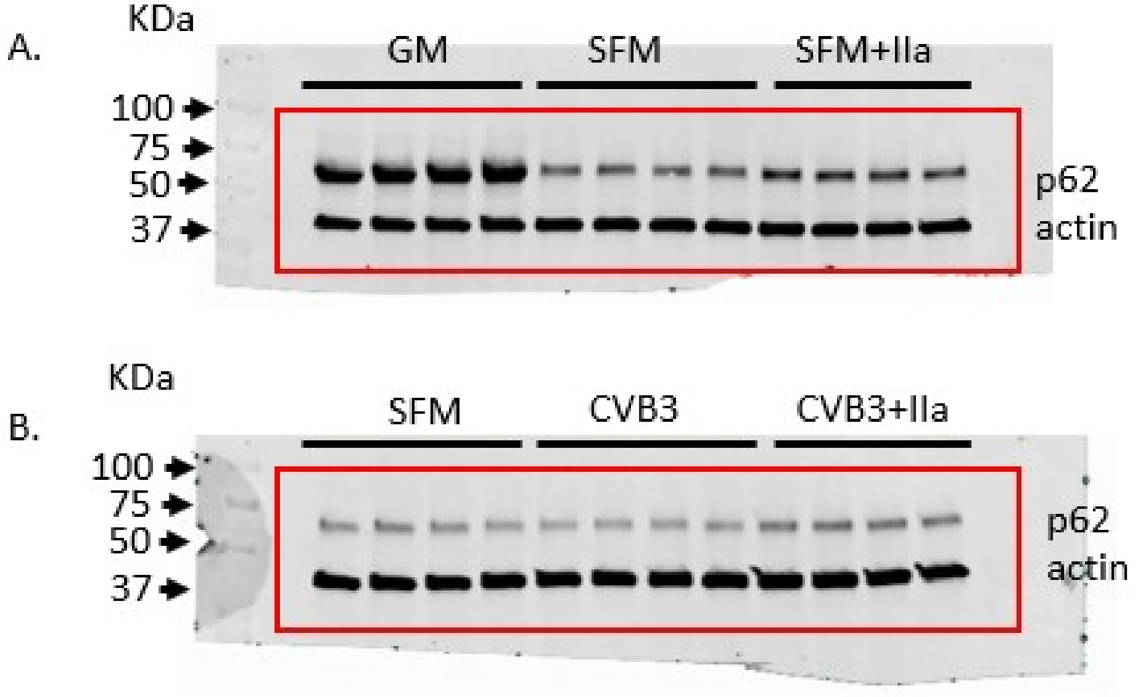

Supplemental Figure 3: Uncut membrane for figure 8 -Thrombin reduces autophagy in serum-starved or CVB3 infected H9c2 cells. A.) Levels of p62 in H9c2 cells with or without serum deprivation in the presence or absence of thrombin (10 nM) for 24 hrs. Membrane shown in figure 8A of main text. **B.)** Levels of p62 in H9c2 cells infected with CVB3 in the absence and presence of thrombin (10nM) for 24 hrs. Membrane shown in figure 8C of main text. Box indicates cropped membrane as shown in figure 8 of the main text.
